# Trend of social media use by undergraduate medical students; a comparison between medical students and educators

**DOI:** 10.1016/j.amsu.2022.104420

**Published:** 2022-08-28

**Authors:** Sumera Nisar, Asim Muhammad Alshanberi, Ahmed Hafez Mousa, Manal El Said, Fatma Hassan, Areeb Rehman, Shakeel Ahmed Ansari

**Affiliations:** a Department of Ophthalmology, School of Medicine, Batterjee Medical College, Jeddah, Saudi Arabia; b Department of Community Medicine and Pilgrims Health Care, Umm Alqura University, Makkah, Saudi Arabia; c College of Medicine and Surgery, Batterjee Medical College, Jeddah, Saudi Arabia; d Department of Microbiology, Theodor Bilharz Research Institute, Giza, Egypt; e Medical Physiology Department, Batterjee Medical College, Jeddah, Saudi Arabia; f University College of Medicine and Dentistry, Lahore, Pakistan; g Department of Biochemistry, School of Medicine, Batterjee Medical College, Jeddah, Saudi Arabia; h Medical Physiology Department, Kasr Al Ainy, Faculty of Medicine, Cairo University, Cairo, Giza, Egypt; i Department of Microbiology, Medicine Program, Batterjee Medical College, Jeddah, 21422, Saudi Arabia

**Keywords:** Medical education, COVID 19, Learning, Communication, Social media

## Abstract

**Purpose:**

Social media (SM) is one of the most powerful tools of communication and learning in the recent era. Different types of information can be shared through these social networking sites in the form of texts, videos, pictures, audios, and references (contacts). Due to the constant increase in the use of these social networking sites in our daily routine life especially during the COVID 19 pandemic, their use in teaching and learning has become inevitable. Social media has immense potential to enhance its role in educational settings. Both the students and educators use it for communication, education, sharing and expressing knowledge, and recreation. Therefore, the present study aims to find out the most frequently used social network sites for learning and easy communication between medical students and educators.

**Objective:**

This study sought to explore the most frequently used social networking sites by the medical students and educators at Batterjee Medical College.

**Methods:**

A cross-sectional study was carried out to assess the trends of usage of SM as an extracurricular way of enhancing learning and teaching experience among medical students and educators in Batterjee Medical College; Saudi Arabia from November 2020 to March 2021.

A pre-validated self-administrated questionnaire was built using Google Drive forms and distributed to medical students and educators via emails and WhatsApp. Convenient sampling was used to collect the data.

**Conclusion:**

Social media has immense potential to enhance its role in educational settings. Students in our study preferred YouTube and WhatsApp for their learning and communication especially, during COVID 19 pandemic. However, to further enhance their utility choosing the right platform, the amount and quality of the information shared to ensure optimal benefit, providing ethical guides, and professional standards for SM use at institutional levels are the few challenges that need to address.

## Highlights

• Our study has also shown the common use of SM by educators and students in the medical field.
• Contemporary and efficient use of social media in medical education cannot be overlooked by educators.
• The students and educators have appreciated the role and availability of social media websites for their easy communication and continuous support for their educational activities during the COVID pandemic.

## 1 Introduction

Social media (SM) is one of the most powerful tools of communication and learning in the recent era [1]. By social media, we mean web tools and different applications designed to enhance knowledge, communication, and instant information sharing [2]**.** Different types of information can be shared through these social networking sites in the form of texts, videos, pictures, audios, and references (contacts). Due to the constant increase in the use of these social networking sites in our daily routine life, their use in teaching and learning has become inevitable [3]. There are many social media sites that are being used by the students and educators for their learning, teaching, clinical skill training, sharing their experiences and communications e.g. YouTube, Facebook, Twitter, Skype, Google, WhatsApp, Wikis, etc. [4]. Although these sites are frequently used by the students and educators but have not been considered a formal instructional tools for learning and teaching [5].

Students use social media to get new information and to communicate effectively with their peers [6]. This method of learning and teaching has become very common and popular among medical students [7]. Learning by social media has grown so wide that it has crossed the boundaries of the classrooms and engages the student in their formal and informal learning [8]. Modern-day curriculum in medical colleges is also promoting and encouraging e-learning [9]. This indirectly relates to the use of social media for learning and empowers students to take charge of their own learning [10].

According to a survey carried out in the united states, 79.4% of medical students are active on online social media for their learning [11]**.** They found it an easy and a simple way of getting information and education material in a format generally acceptable for the new generation. This survey also showed that the young medical students are more responsive and receptive to these online teaching-learning tools even in the absence of any prior knowledge or exposure to such educational methods. This also has shifted the mode of learning from printed textbooks to electronic media. This study has also proved the preference of the medical students to receive educational material and information online.

Although many educators have adopted new trends and technologies in their daily teaching strategies still there is a big number of educators struggling or resisting the new trends of social networking in medical education. The faculty development programs can help to bridge this technology gap between the students and the faculty. Social media is being widely used by students all over the world including in Saudi Arabia but its formal use in medical schools is not clear. The students are really keen and enthusiastic to learn by using social media/networking tools but the response and willingness of the educators need to be explored.

The main aim of the study is to identify the most commonly used social media/networking sites for learning and communication by the medical students and medical educators in a local medical school of Saudi Arabia and to know the gap between the students and the educators in using social media tools for their learning.

Also, to identify the perception of medical students and educators about knowing the guidelines or ethical principles of social media use in medical education. This study was done in compliance with the STROCSS 2021 criteria [34].

## 2 Materials and methods

A cross-sectional study was carried out to assess the trends of usage of SM as an extracurricular way of enhancing learning and teaching experience among medical students and educators in Batterjee Medical College; Saudi Arabia from November 2020 to March 2021.

A pre-validated self-administrated questionnaire was built using Google Drive forms and distributed to medical students and educators via emails and WhatsApp. Convenient sampling was used to collect the data. The questionnaire was initially sent to 150 health educators and students. A total of 128 participants, 101 medical students, and 27 medical educators responded to the survey. The response rate was 85.3%. The missing data were excluded from the study.

The study was approved by Batterjee medical college (BMC), Jeddah, Saudi Arabia research and ethical review committee (RES-2020-0061). Informed verbal and written consent were taken from the students.

The study included medical students from preclinical and clinical years of medicine (M1-M5) and medical educators e.g. professors, associate professors, assistant professors, and lecturers at BMC. Non-medical students and interns were excluded.

The questionnaire consisted of four sections: the first section included questions on student's and educator's demographics (age, gender, year of study, academic post-graduate ranking, and years of experience). The second part was about patterns of SM (usage of SM, frequency, type of SM, and preferred platform). The third part included questions on the use and influence of SM on learning and teaching (influence on education, communication, and collaboration). Finally, participants were also invited to suggest comments that further supported our study. The questionnaire consisted of a Likert-type scale questions (where 1 = strongly disagree, 2 = disagree, 3 = natural, 4 = agree, and 5 = strongly agree), multiple choices, yes/no, and short answer questions.

Analyses was performed with SPSS “statistical package for social sciences” (version 20). Descriptive analyses of the data were represented in tables and graphs in terms of percentages and used to estimate the frequency and prevalence of using SM for learning purposes.

## 3 Results

### 3.1 General information on participation

The total number of respondents was 101 medical students and 27 educators in the survey. Females represented 64.4% (n = 65) of the total participants. The majority of participants belonged to the clinical sciences (60.4%; 61/101). Demographic details of the participants both medical students and educators are illustrated in Table 1.

**Table 1:** Demographic Details of Medical students and educators Participants.

Characteristic	Medical Students No. (%)	Educators No. (%)
**Gender**		
Male	36 (35.6)	15 (55.6)
Female	65 (64.4)	12 (44.4)
**Age**		
Range	18–24	20–55
Average ± SD	24±	38.9±
**Medical year**		
Medical phase (M1, M2 and M3)	40 (39.6)	**-**
Clinical phase (M4 and M5)	61 (60.4)	**-**
**Academic post-graduate ranking**		
Professor	**-**	[5]18.5
Associate Professor	**-**	10 (37.1)
Assistant Professor	**-**	6 (22.2)
Lecturer	**-**	6 (22.2)
**Years of experience**		
Range	**-**	1.5–20
Average ± SD	**-**	11.35±

### 3.2 Pattern and purposes of SM use for educational purposes

The majority of students and educators had a presence on social networking sites in 89.1% (90/101) and 88.8% (24/27) respectively. With regard to utilizing social networking sites for learning, 69.3% (n = 70) of the students and 77.8% (n = 21) of the educators believed that it is beneficial.

The most common websites used by students for their learning were YouTube (92.1%, n = 93) and Blackboard (63.3%, n = 64) as shown In Fig. 1A, Fig. 1BA and 1B. However, educators preferred using Blackboard (59.2%, n = 16) as shown in Fig. 2A, Fig. 2BA and 2B. WhatsApp was the second most commonly used social media by both students (71.2%) and educators (62%) for easy communication respectively (Fig. 1A, Fig. 1B, Fig. 2A, Fig. 2B). Most students (90.6%) used SM for learning for at least 1 h per day or more. The preferred device to use when students attend sessions or educators teaching students was the laptop by 57.4% (n = 58) and in 62.9% (n = 17) respectively followed by the tablet.

**Fig. 1A:**
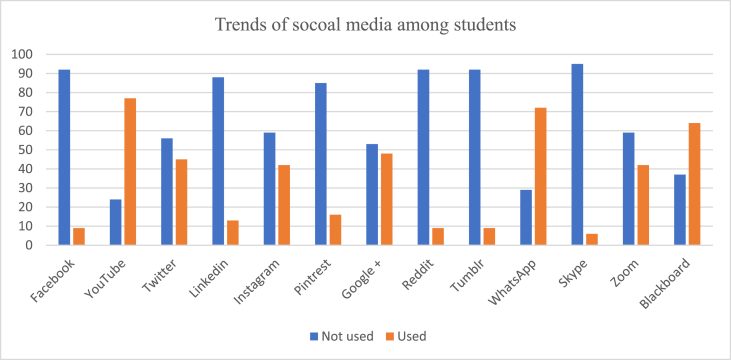
Trend of social media used in medical education by medical students.

**Fig. 1B:**
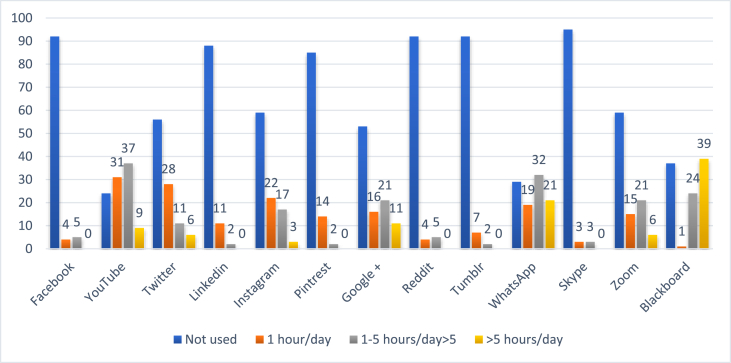
Trend of social media used in medical education by medical students.

**Fig. 2A:**
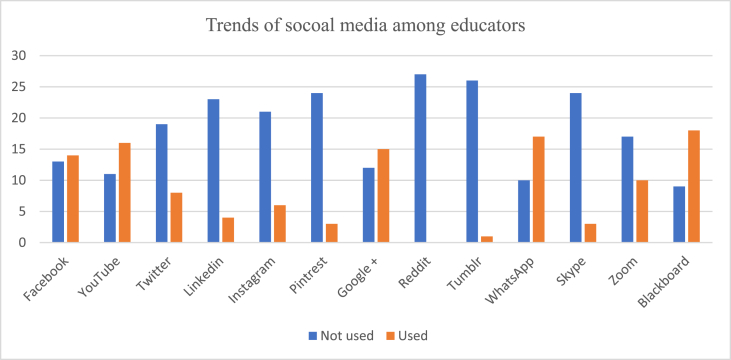
Trend of social media used in medical education by educators.

**Fig. 2B:**
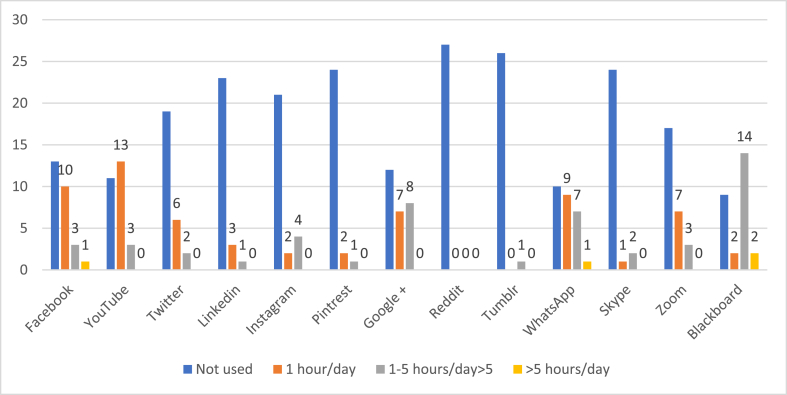
Trend of social media used in medical education by educators.

**Fig. 3:**
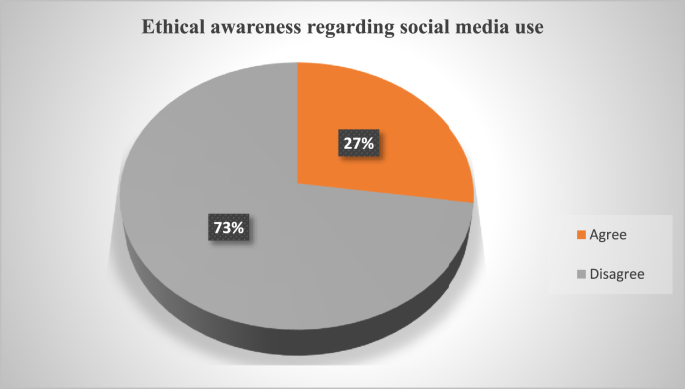
Ethical guidelines awareness regarding social media use.

Moreover, 73% of the study participants were not aware of the ethics for SM use (Fig. 3).

## 4 Discussion

The advancement in technology has transformed the educational process and communication among the students and educators, especially during the recent experience of the COVID 19 pandemic [12]. The ease of communication has made it an excellent mode for student interaction and information sharing [13]. Also, the pandemic led restrictions, especially in medicine have favored the use of social media for academic communication and learning. Many social media sites particularly Facebook, WhatsApp, YouTube, and Twitter have been successfully used as an instructional aid in teaching and learning and have influenced the educational process worldwide [14]. Although, the motive of these sites is to engage people in social activities and communication; the students found it very handy to share and disseminate knowledge and their experiences. Due to the latest trend of social media use in education, these sites are also rapidly improving in delivering educational content, especially for medical educators and students [15].

Our study has also shown the common use of SM by educators and students in the medical field. Of all participants, students and educators had a presence on social networking sites which is 89.1% (90/101) and 88.8% (24/27) respectively. The study also showed the propensity of clinical years students toward the frequent and regular use of SM. Health care workers and medical students need continuous updates on their knowledge and skill throughout their professional careers. Many medical students consider SM as an efficient and effective tool for enhancing their clinical and problem-solving skills [16]. Clinical training is an essential component of medical education which has been immensely affected by the COVID 19 pandemic. Most of the students and educators in our study have explored and experienced the exquisite use of SM during that difficult time to support their learning [17]. Also, many other studies have explored the effectiveness of social media in training the clinical skills of medical students as well as in promoting or recruiting patients for clinical skill training and trials [18,19]. The students and educators have appreciated the role and availability of social media websites for their easy communication and continuous support for their educational activities during the COVID pandemic [20]. They also found it useful in getting updated health information [21]. Although despite all its benefits, for all these training and interactions through social media, care must be taken by providing enough awareness about the risks of SM use. The content validity and accuracy for medical students on SM is a rising concern [22].

The most commonly used platform used by students in our study was YouTube (92.1%, n = 93). Most of the students found it useful in understanding the difficult concepts and helpful in relating basic science knowledge with the clinical. The use of social media especially YouTube helped medical students explicitly to overcome the learning challenges during the COVID pandemic [23]. They found it a useful resource for health and educational information videos augmenting their knowledge [24]. Moreover, certain factors unique to YouTube make it preferred by students as searching for a specific topic on YouTube is easier and doesn't necessitate any account compared to regular formal academic websites [24]. Many studies have also explored the content appropriateness on YouTube and the provision of reliable content [25], yet the source of the training videos must be confirmed before use [26].

However, many students (73%) in our study were not very well aware of the ethics of SM use. Despite a growing number of SM used in medical education, ambiguity about the content appropriateness and professional behavior of the students prevails. A scarcity of clear SM usage guidelines and policies to protect the students and health workers is also a concern [27].

On the other hand, educators preferred using Blackboard (59.2%, n = 16). They found it an easy and beneficial mode of communication with the students for education activities. They were well aware of their student's needs and use of social media for these educational activities. Many institutes in Saudi Arabia have used Blackboard as an alternative instructional and communication tool during COVID 19 pandemic. All the available features are well designed for educational purposes but need some training workshops to learn their efficient use [28,29]. However, most of the educators in our study were not aware of the ethics of social media use.

A huge number of students and educators preferred WhatsApp as another alternative and easiest way of communication with their colleagues from the same and other institutes. WhatsApp is the most popular mode of communication worldwide but its role in medical education still needs further exploration [30]. Although some studies have recommended WhatsApp use in medical education due to its easy, interactive, and instant role in preparing the students for their day to day learning activities [31,32] its educational effectiveness lacks conclusive evidence [33].

Overall, the study has explored the beneficial and effective engagement of both students and educators on social media for their easy communication and learning. YouTube, Blackboard, and WhatsApp are the most frequently used social network sites by the participants of our study. However, despite the frequent use of the social networking sites by both students and educators, none of them were very well aware of the ethics and guidelines of SM use.

## 5 Limitations and implications

A limitation of our study is that it was limited to a single institution. More institutes could have been added to get more extensive data. Additionally, we highly recommend that additional in-depth assessment and comparison between different subsets to be done.

## 6 Conclusion

Social media has immense potential to enhance its role in educational settings. Both the students and educators use it for communication, education, sharing and expressing knowledge, and recreation. Their contemporary and efficient use cannot be overlooked by educators. To use them to their fullest; the material posted needs evaluation to be accurate, concise, and engaging. Moreover, choosing the right platform, the amount and quality of the information shared to ensure optimal benefit, providing ethical guides, and professional standards for SM use at institutional levels are the few challenges that need to address.

## List of abbreviations

SNSs: Social networking sites; SM: Social media.

## Ethics approval

Ethical approval has been given by the Institutional Review Board (IRB) of our institution, Batterjee Medical College, Jeddah, Saudi Arabia

## Source of funding

No funding was provided for this study.

## Author contribution

Drafting of the manuscript: Ahmed Hafez Mousa, Asim Muhammad Alshanberi, Sumera Nisar, Fatma Hassan, Manal El Said, Areeb Rehman, Shakeel Ahmed Ansari. Critical revision of the manuscript for important intellectual content: Ahmed Hafez Mousa, Asim Muhammad Alshanberi, Sumera Nisar, Fatma Hassan, Manal El Said, Areeb Rehman, Shakeel Ahmed Ansari.

## Registration of Research Studies

1. Name of the registry
2. Unique Identifying number or registration ID
3. Hyperlink to your specific registration (must be publicly accessible and will be checked):

## Consent

The study was approved by Batterjee medical college (BMC), Jeddah, Saudi Arabia research and ethical review committee (RES-2020-0061). Informed verbal and written consent were taken from the students.

## Guarantor

Ahmed Hafez Mousa, corresponding author of the manuscript, accept full responsibility for the work and the conduct of the study, had access to the data, and controlled the decision to publish.

## Availability of data and material

Data supporting the findings of the study are available upon request from the corresponding author.

## Declaration of competing interest

All authors declare that they have no conflict of interest.
